# Effects of Stoichiometric Variations in L-Arginine-Cured Epoxy Resins

**DOI:** 10.3390/polym17223089

**Published:** 2025-11-20

**Authors:** Melissa Walter, Dennis Gibhardt, Bodo Fiedler

**Affiliations:** 1 Hamburg University of Technology, Institute of Polymers and Composites, 21073 Hamburg, Germany; dennis.gibhardt@tuhh.de (D.G.); fiedler@tuhh.de (B.F.); 2 United Nations University Hub on Engineering to Face Climate Change at the Hamburg University of Technology, United Nations University, Institute for Water, Environment and Health (UNU-INWEH), 21073 Hamburg, Germany

**Keywords:** sustainability, biomolecules, thermo-mechanical properties, fourier transform infrared spectroscopy, network structures, reaction mechanism

## Abstract

For the purpose of reducing environmental and health risks in the production of fibre-reinforced polymers, biomolecules are increasingly examined as alternative resources. For example, amino acids can serve as curing agents for epoxy resins. However, their particular appearance and possible reactions differ from those of conventional hardeners. To find a performance-optimised mixing ratio, it is relevant to know how deviations in the mixing ratio affect the reactions that take place and the resulting thermo-mechanical properties. Consequently, in this study, eleven mixing ratios of L-arginine-cured DGEBA without a catalyst or accelerator were investigated optically, thermo-mechanically, and via FTIR analysis. Based on the theoretical stoichiometric ratio, a wide range of good thermo-mechanical properties between stoichiometric ratios of R = 0.8 and R = 1.0 could be determined. However, this study led to an extension of a possible reaction mechanism for the curing of epoxides with amino acids, particularly L-arginine, postulating the thermo-induced deprotonation of α-NH_3_^+^ groups, etherification as part of successful crosslinking, and the unfavourable reactivity of the guanidium group in the case of L-arginine, shifting the optimal to slightly sub-stoichiometric configurations.

## 1. Introduction

Biomolecules as matrix components for use in fibre-reinforced polymers can not only reduce geopolitical dependencies and avoid scarce resources but also decrease the number of risks posed to the environment and health. Amino acids have been successfully investigated as sustainable curing agents for epoxy resins [1,2,3].

**Figure 1:**
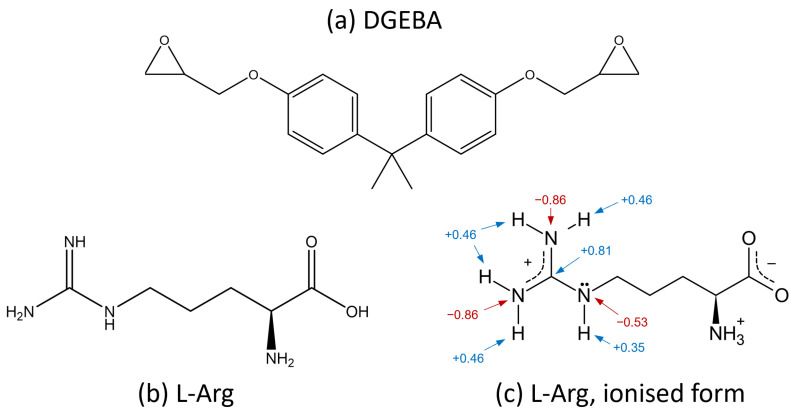
Chemical structures of (**a**) DGEBA, (**b**) L-Arg, and (**c**) L-Arg in its ionised form. Partial charges in the side chain obtained from [4].

In previous studies, L-arginine (L-Arg) has shown outstanding potential because of its high proportion of potentially active hydrogen atoms. So far, the reaction mechanism of Bisphenol A diglycidyl ether (DGEBA) with L-Arg has not been fully and conclusively understood. However, the functional groups present in L-Arg indicate significantly more possibilities to react with epoxies or other L-Arg molecules than standard curing agents. Figure 1 shows the chemical structure of DGEBA (a), L-arginine (b), and its crystalline ionised state, which contains a mesomerically stabilised guanidinium ion (c) [4].

The fact that individual functional groups are difficult to access, for example, due to steric hindrance, as well as possible changes in the network structure due to an excess of one of the two components or changes in molecular interactions (e.g., hydrogen bonds), makes it relevant to investigate various mixing ratios to exploit the full potential and limits of arginine-cured epoxy resin for use as a matrix material in fibre-reinforced polymers and to gain further knowledge of the reaction mechanism and network structures.

According to Ullmann et al. [5], amine curing agents are used in near stoichiometric ratios to obtain performance-optimised polymers. Several studies have shown that the stoichiometric ratio of epoxy resins and their curing agents has a significant effect on chemical and physical properties [6,7,8]. The use of excess amine will result in unreacted amine-terminated dangling chain ends and reduced crosslinking, resulting in tougher but less moisture- and chemical-resistant polymers. Nevertheless, Li et al. [9] reported that systems with a volume of over-stoichiometric curing agents resulted in an increase in tensile strengths and moduli and a decrease in the thermal expansion coefficient. Furthermore, the authors found that over-stoichiometric mixtures can improve cryogenic properties [9]. Morsch et al. [7] confirmed that epoxy resins changed from homogeneous internal nanostructures to ones with nodular morphology characteristics in the presence of excess epoxy. Although amine-rich mixtures also showed a greater deformation capacity, D’Almeida et al. [10] and Tavares et al. [11] reported the formation of tight macromolecular structures that result in brittle behaviour in epoxy-rich mixtures. Furthermore, the stoichiometric ratio had a great influence on the room-temperature ageing process of epoxy resins. Among other reasons, D’Almeida et al. [10,12] identified the recrystallisation of unreacted epoxy monomers as a reason for the increased brittleness of epoxy-rich systems with ageing treatment, while amine-rich configurations had more stable mechanical properties.

Most studies are conducted with liquid curing agents. However, particulate curing agents such as amino acids might especially show differences. Some studies investigated changes in the mixing ratio for amino acid-cured epoxies: With an increasing amount of L-Tyrosine as a curing agent, Rothenhäusler et al. [13] found improved fracture toughness due to crystal formation, while, e.g., the glass transition temperature only changed slightly. Li et al. [1] investigated L-Tryptophan-cured DGEBA with three different mixing ratios and found differences in reaction enthalpies and glass transition temperatures with the maximum values for theoretically sub-stoichiometric ratios.

To the best of the authors’ knowledge, no studies on stoichiometry variation on arginine-cured epoxides have been published yet. Because arginine differs significantly from conventional amine curing agents from a chemical point of view, key questions arise that will be addressed in this study. Firstly, it is relevant to investigate which mixing ratio leads to performance-optimised L-arginine-cured DGEBA, because this is not necessarily the case for the stoichiometric ratio, e.g., anhydrides are normally used with sub-stoichiometric ratios of 0.5 to 0.85 [5] due to significant epoxy homopolymerisation. Secondly, it is valuable to figure out which mixing tolerances exist without significantly changing the investigated properties. Thirdly, it is beneficial to examine whether a change in the mixing ratio of arginine-cured epoxy can contribute to a better understanding of the reaction mechanism if the excess of one component favours or prevents possible reactions and whether experimental methods can capture these changes.

## 2. Materials and Methods

### 2.1. Materials and Stoichiometric Variations

This study was carried out on an epoxy system consisting of DGEBA-based resin 827 (Epikote™ 827, Westlake, Houston, TX, USA) and the amino acid L-Arg (L-Arginine, Buxtrade GmbH, Buxtehude, Germany) as a biobased curing agent. As described in [3], the stoichiometric mixing ratio was theoretically calculated using the amine hydrogen equivalent weight (AHEW) of the amino acids. Therefore, the molecular weight of L-Arg (M_W_ = 174 g/mol) was divided by seven, the number of active hydrogen atoms in the molecular structure. The stoichiometric mass ratio (1) was calculated considering the epoxy equivalent weight (EEW) of 827 (EEW = 182 g/mol) with m_i_ as the respective component mass:

(1) $$\frac{m_{827}}{m_{L-\mathrm{Arg}}}=\frac{100\;g}{100\;g\;\cdot \;\frac{\mathrm{AHEW}}{\mathrm{EEW}}}=\frac{100\;g}{100\;g\;\cdot \;\frac{25\;g/\mathrm{mol}}{182\;g/\mathrm{mol}}}=\frac{100\;g}{13.74\;g}$$

In the following, this theoretically calculated stoichiometric mixture is referred to as R = 1.0. In this study, the amount of L-Arg is changed between 50% (R = 0.5) and 150% (R = 1.5) of the stoichiometric amount. Table 1 shows their weight-related mixing ratios.

### 2.2. Manufacturing and Testing

The amino acid is dispersed through a 120E three-roll mill (EXAKT Advanced Technologies GmbH, Norderstedt, Germany) as described in [3]. Dispersions were tested rheologically with an ARES rheometer (TA Instruments Inc., New Castle, DE, USA) in plate–plate mode according to DIN EN ISO 3219 [14] (20–50 °C, 2 K/min, plate diameter: 40 mm; gap: 0.5 mm; frequency: 5 Hz; shear strain: 10%). After degassing, plates of all configurations were manufactured by a casting process with the curing profile determined in [3] for stoichiometric mixtures via thermokinetic analysis (20 °C to 167 °C (4 K/min); 167 °C (60 min); 167 °C to 180 °C (0.05 K/min); 180 °C (70 min); 180 °C to 20 °C (−0.5 K/min)).

Since a qualitative comparison between the introduced configurations is made, the geometry of the specimen according to [15] was chosen due to their known accurate and reproducible performance in tensile testing. Furthermore, the geometry allows for resource-saving manufacturing and the use of similar specimen designs for viscoelastic analysis (DMA-T_g_ measurements). The influence of the specimen volume, strain rate variations, etc., was not considered. Dogbone-shaped samples were milled with a EUROMOD^®^-MP (Isel Germany AG, Eichenzell, Germany), conditioned for 24 h at 40 °C under vacuum, and tested on a GABO EPLEXOR^®^ 500 N (Erich NETZSCH GmbH & Co. Holding KG, Selb, Germany) in static tensile tests at 1 mm/min (5% strain/min) until failure. Furthermore, dynamic tests were performed on the same machine. To estimate trends in crosslink density, $\nu_{C}$ (Equation (2) [16]) was evaluated for R = 0.5, R = 0.9, and R = 1.5. To obtain $\nu_{C}$ in the unit mol · cm^−3^, the storage modulus $E^{\prime }$ in the rubbery plateau is inserted in MPa and $T_{g}$ in K.

(2) $$\nu_{C}=\frac{E_{T_{g}+40K}^{\prime }}{3\;\cdot \;8.314\frac{J}{\mathrm{mol}\;\cdot \;K}\;\cdot \;\left(T_{g}+40K\right)}$$

Glass transition temperatures (T_g_s) were determined in accordance with DIN EN ISO 6721-1 [17] via onset analysis (4 × 8 × 50 mm^3^, 1 Hz, 2 K/min, 20 °C to 150 °C). Additionally, T_g_s were determined using DSC 204 F1 Phoenix (Erich NETZSCH GmbH & Co. Holding KG, Selb, Germany) according to DIN EN ISO 11357-2 [18] via midpoint analysis. A heating rate of 10 K/min was defined for two heating runs (max. temperatures: 1. 180 °C; 2. 200 °C). Furthermore, uncured resin was investigated via DSC, and the reaction enthalpy and peak position were determined for the mixing ratios R = 0.5, R = 1.0, and R = 1.5 (10 K/min, max. temperature: 300 °C).

### 2.3. Fracture Surface Analysis

Fracture surfaces were analysed using a VHX-6000 digital microscope (Keyence Corporation, Osaka, Japan). Additional scanning electron microscopy (SEM) images were acquired with a SUPRA 55VP (Carl Zeiss AG, Oberkochen, Germany) using secondary electron (SE) detection with an accelerating voltage of 3 kV. All samples were provided with silver conductive paint at the edge of the sample carrier and vaporised with gold for 30 s at 40 mA with a BALTEC SCD 050 SPUTTER (BALTIC Präparation e.K., Wetter, Germany), creating an electrically conductive layer with a thickness of a few nanometres on the surface.

### 2.4. Fourier Transform Infrared Spectroscopy

FTIR measurements in the MIR range (4000 to 500 cm^−1^) are performed on a Tensor II (Bruker Corporation, Billerica, MA, USA) in attenuated total reflectance (ATR) using a MIRacle™ Single Reflection Horizonal ATR Accessory from PIKE Technologies (Fitchburg, WI, USA). The resolution is set to 2 cm^−1^, and for background and sample spectra, the mean values of 40 measurements are used. The processing of raw data includes a baseline correction and normalisation of the spectra on the constant aromatic peak (1605 cm^−1^, C=C stretching). Gaussian fitting is used to determine the positions of the peaks.

## 3. Results and Discussion

### 3.1. Optical Examination

**Figure 2:**
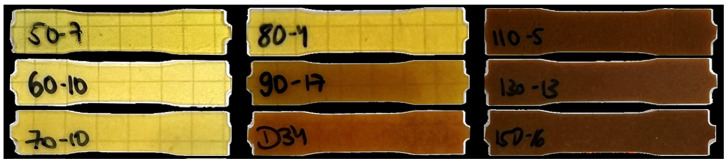
Photographs of test specimens with different mixing ratios. Labelling x-y with x as L-Arg content in wt.-% of the theoretical stoichiometric amount and y as numbering for traceability. Stoichiometric configuration from plate D.

Arginine-cured epoxies become increasingly opaque with an increasing number of L-Arg particles (see Figure 2). This is due to increased light diffraction in the agglomerates of particles. At up to 80% of the theoretically stoichiometric amount of L-Arg, the specimens are still yellow-transparent, and at 90% and 100%, they become increasingly brownish. In over-stoichiometric mixtures, the background is no longer recognisable. Within the over-stoichiometric mixtures, no significant differences with further increases in arginine quantity are observable.

### 3.2. Rheological Characterisation

**Figure A1:**
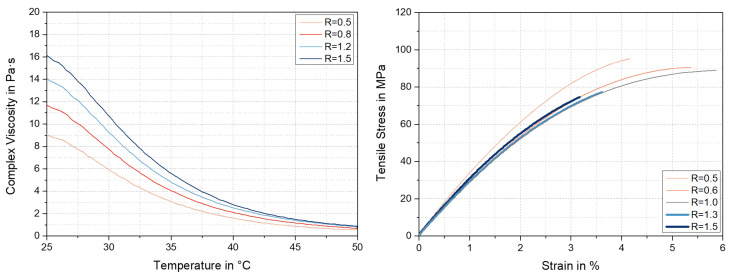
Temperature-dependent viscosity (**left**) and representative stress–strain curves (**right**) for selected mixing ratios.

Temperature-dependent viscosities for selected mixing ratios are shown in Figure A1 in Appendix A. Because of the higher amount of the solid component, an increase in the stoichiometric ratio results in higher viscosities at RT, whereas the differences diminish with higher temperatures. In detail, the complex viscosities are between 9 Pas (R = 0.5) and 16 Pas (R = 1.5) at 25 °C and approximately 1 Pas for all configurations at 50 °C onwards.

### 3.3. Fourier Transform Infrared Spectroscopy

The thermograms of the DSC measurements carried out do not show any exothermic peaks after curing. This indicates that the exothermic reactions took place completely during the curing process. However, if one component is missing, the other cannot react completely and is present partially reacted or unreacted. FTIR measurements are performed to gain deeper knowledge of the reactions that occur in the different configurations.

**Figure 3:**
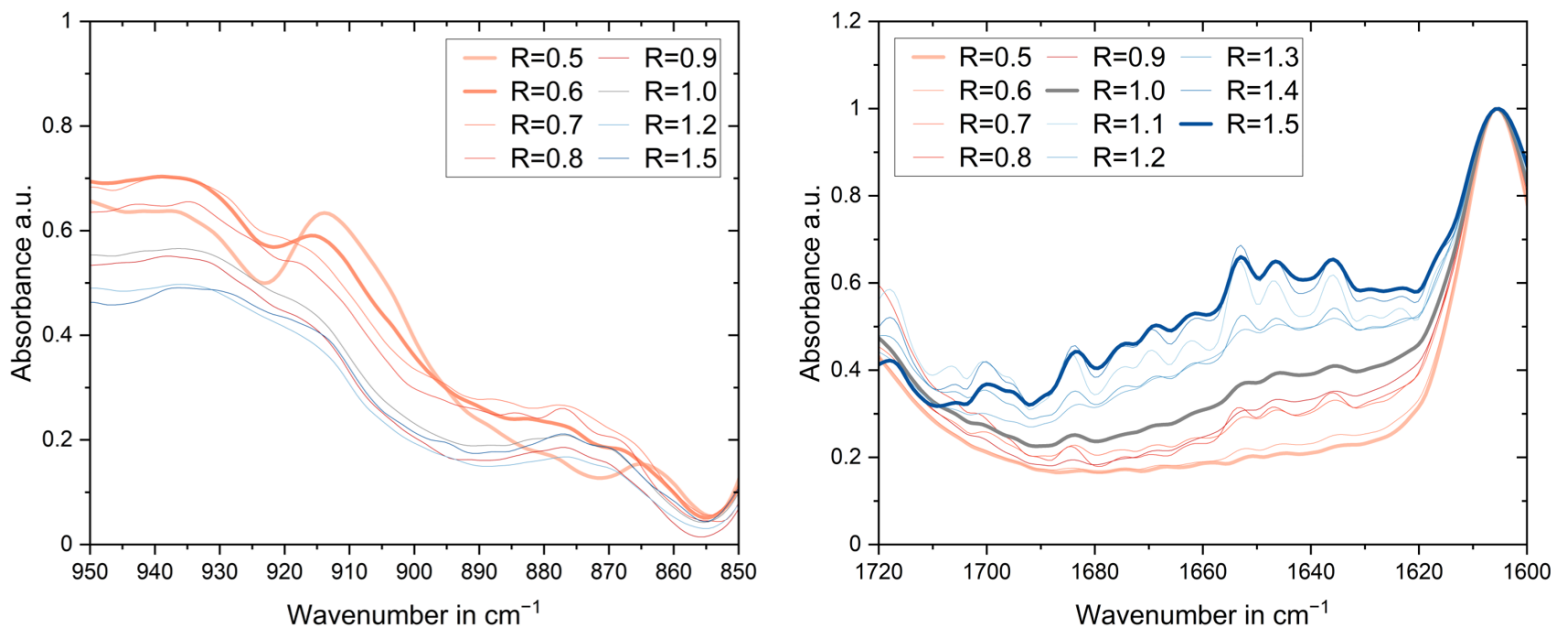
Normalized FTIR spectra of all stoichiometric ratios between 950 and 850 cm^−1^ (**left**) and 1720 and 1600 cm^−1^ (**right**).

Figure 3 (left) displays the absorbance data in the range of the epoxy peak (≈910–915 cm^−1^) [19,20]. The severely sub-stoichiometric ratios with 50% and 60% of the theoretically stoichiometric amount of L-Arg show a significant epoxy peak. For the other sub-stoichiometric ratios, the left-over epoxy is still visible in the form of a shoulder in this wavenumber range. All other configurations do not show specific epoxy peaks. The shape and heights of the peaks correspond to expectations: For R = 0.5, the peak is larger as the epoxy groups remain. This observation is in line with the outstandingly different results of the thermo-mechanical characterisations for R = 0.5 and R = 0.6 compared to the other theoretically sub-stoichiometric ratios discussed later.

Figure 3 (right) shows the spectra between 1720 cm^−1^ and 1600 cm^−1^. In this range, there are various vibrations of C=N groups, which can be present in acyclic arginine (1690–1640 cm^−1^) and conjugated arginine (1660–1630 cm^−1^) and as imines (1640–1633 cm^−1^). The vibrations of primary amides (1650–1620 cm^−1^) are also present. These can be formed during the reaction of two arginine molecules through the formation of peptide bonds. According to Li et al. [1], a peak at 1665 cm^−1^ corresponds to NH_3_^+^ asymmetric deformations. Primary and secondary amines (1650–1590 cm^−1^) are also reported in this wavenumber range. These cannot be detected for the configurations R = 0.5 and R = 0.6, which is consistent with the epoxy groups discussed above that are still present in the system. From R = 0.7 onwards, an increase in the spectrum in this wavenumber range is recognisable as a superposition of the oscillations mentioned. This increase rises with an increase in the arginine content, whereby a disproportionately high enhancement is visible for over-stoichiometric mixtures (R ≥ 1.1). This supports the forthcoming discussion that a significant drop in many of the thermo-mechanical properties analysed is observed from R = 1.1 upwards.

**Figure 4:**
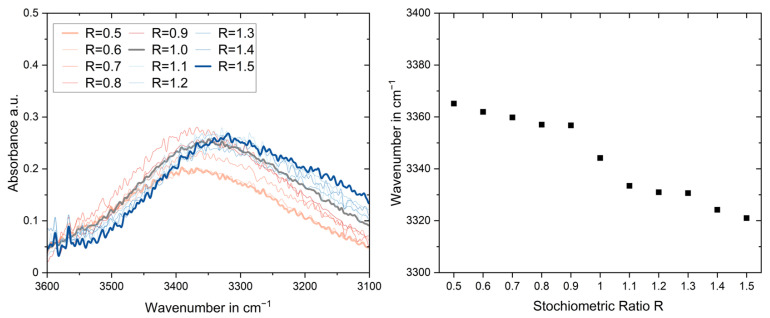
Peak shift between 3600 cm^−1^ and 3100 cm^−1^ with stoichiometric variation.

A significant change for over-stoichiometric configurations is also visible in the peak position between 3600 and 3100 cm^−1^ (Figure 4). Both a clear peak shift and a change in peak height with an increase in the arginine content are recognisable.

In this range, the peaks of several oscillations overlap, the exact naming and position of which sometimes shows slight deviations in the literature. According to Mora et al. [21], there are peaks in this region that can be assigned to different types of hydrogen bonds. The wavenumber of hydrogen bonds, e.g., (-OH—HO-) or (-OH—HN-), would be lower in molecules and prepolymers than in networks, which matches the peak shift shown in Figure 4, as more molecules are present unreacted when more arginine is present in the system.

Furthermore, according to Kolev [22], a peak at 3288 cm^−1^ is characteristic of amides. As described above, these are likely to form more when more L-Arg is present in the system. This may be another reason for the shift in the superimposed peak to lower wavenumbers for over-stoichiometric configurations. Li et al. [1] reported a broad peak between 3200 cm^−1^ and 2700 cm^−1^ corresponding to NH_3_^+^.

**Figure A2:**
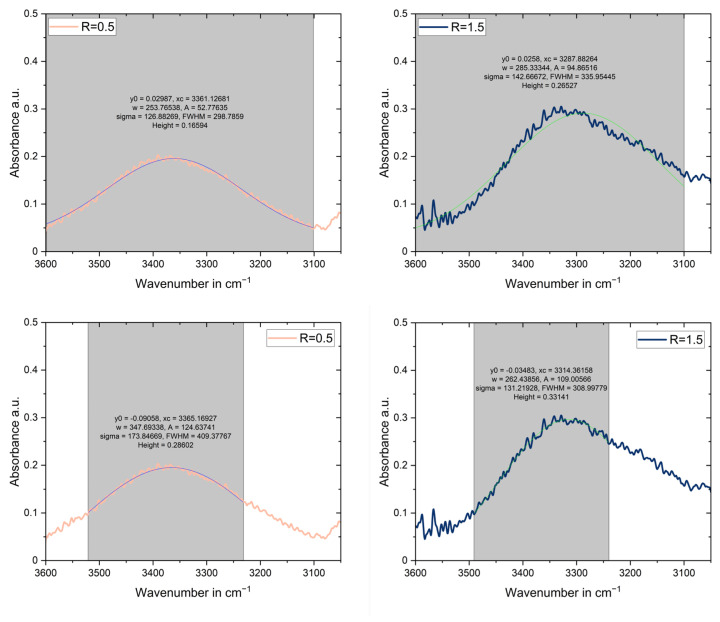
Determination of peak positions via Gaussian fitting to evaluate occurring peak shift. Exemplary for R = 0.5 and R = 1.5. (**Top**) Complete peak range as boundary. (**Bottom**) Final boundaries for best fit of asymmetric peaks.

The peak shift is shown in Figure 4 (right) over the stoichiometric ratio R. The theoretical stoichiometric ratio R = 1.0 represents a turning point in this curve. The position of the peaks is determined using Gaussian fitting, as shown in Figure A2. It is noticeable that the peaks for low L-Arg ratios are symmetric so that the Gaussian fit fits over a wide range of the peak. This is no longer the case for higher stoichiometric ratios; strong asymmetry can be observed, which can be explained by a significant increase in an additional peak at lower wavenumbers (e.g., remaining NH_3_^+^, amide formation, intermolecular hydrogen bonds). For this reason, the limits were adjusted for the comparison of the peak maximum so that the maximum of the peak could be visualised with the Gaussian fit.

An increase in peak height has also been reported to be an indicator of an increase in the number of hydrogen bonds [23]. A quantification evaluation is omitted, as the signal strength of the ATR measurements should be treated with caution. However, qualitatively, the peak height is the lowest for R = 0.5 and R = 0.6, which is consistent with Figure 3 (left), indicating that there is no significant amount of amide and -NH bonds for the formation of hydrogen bonds for these configurations.

### 3.4. Mechanical Characterisation

**Figure 5:**
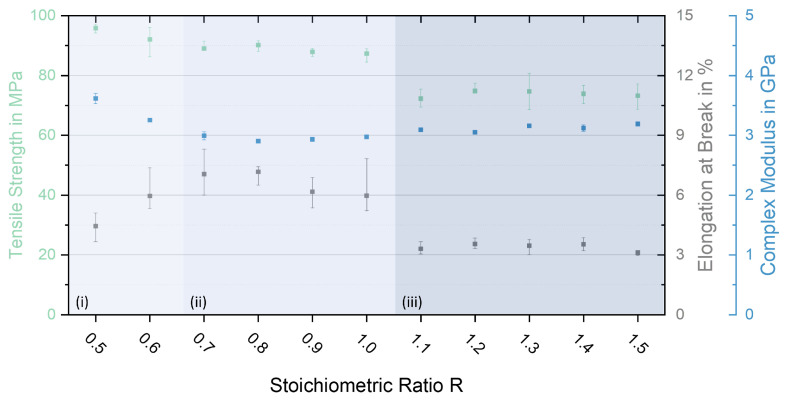
Tensile strength, elongation at break, and elastic modulus for all investigated mixing ratios and categorisation into three zones (i–iii).

Figure 5 displays the tensile strength, the elongation at break, and the elastic modulus for all stoichiometric ratios tested determined at room temperature. Furthermore, the corresponding stress–strain diagram is given in Figure A1 (Appendix A). The achievable tensile strengths cover a range from about 70 MPa to 95 MPa and follow a clear dependence on stoichiometry. Consequently, the range that can be set encompasses an order of magnitude of 25%. While the stiffness remains largely unaffected, the maximum elongation at break is the most sensitive to changes in the stoichiometry of the L-Arg-cured epoxy. Differences of more than 50% occur, whereby the most brittle configurations are found for both extremes, significantly over- and sub-stoichiometric mixtures (Table A1). Young’s moduli were also determined in quasi-static tensile tests. The corresponding results are shown in the Appendix A. From the results obtained, it is evident that the investigated stoichiometry range can be divided into three areas (i–iii). The best mechanical properties in the combination can be observed very consistently for a large range of R = 0.7–1.0. (ii). If the proportion of the particulate curing agent is further increased (iii), the stiffness remains, while the elongation at break drops dramatically. This also leads to a significant reduction in tensile strength, as the specimens break before reaching a yield plateau. Taking into account the propensity for the agglomeration of arginine particles, it can be assumed that an increasingly defect-driven failure occurs for over-stoichiometric mixtures. Here, the non-reacted L-Arg particles serve as imperfections in the network and favour crack initiation. In the investigated range of over-stoichiometric mixing ratios, the L-Arg content only differs by approximately 5 g per 100 g of DGEBA (see Table 1). For occurring failure initiation, it seems that conceivable differences in agglomerate size or distribution have no measurable influence in these relatively small quantities, which might be one reason that no significant decrease in properties is recognisable.

**Figure 6:**
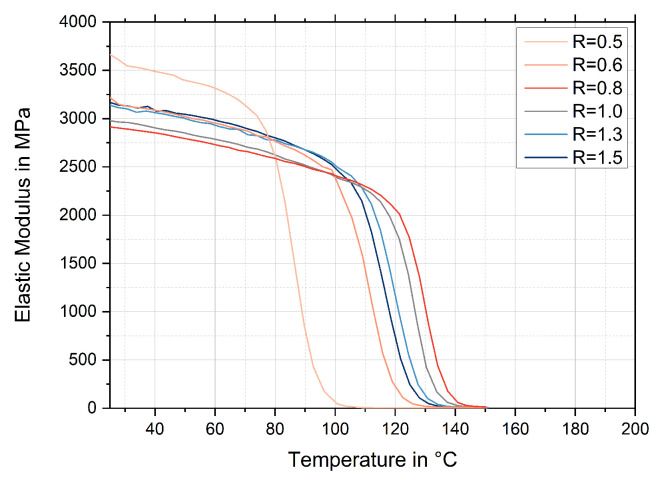
Temperature-dependent elastic modulus for relevant configurations.

For the considerably sub-stoichiometric configurations R = 0.5 and R = 0.6 (i), the failure behaviour also tends to be more brittle. Although stiffness and strength are increasingly high and the highest in comparison with all investigated ratios, the elongation at break progressively decreases. From a purely mechanical point of view, the configurations with the greatest epoxy excess (i) would even be favoured. An explanation for this unintuitive behaviour can be found by taking into account the viscoelastic response, as shown in Figure 6. Here, an analogy to incompletely but stoichiometric cured thermosets can be made. In detail, mechanical properties at room temperature have been reported regularly as not simply following a monotonous trend with an increasing conversion rate. Even though one would expect that a higher degree of crosslinking in a fully (or stoichiometrically) cured thermoset should lead to better mechanical properties, most properties such as stiffness, strength, fracture toughness, creep resistance, etc., show their maximum values at conversion rates of approx. 75–90% [24,25]. Further and closer crosslinking then merely raises the glass transition temperature and improves the properties at higher temperatures, while the mechanical properties at room temperature are significantly worsened. The reason for these surprising effects are the differences in the free volume of the corresponding networks at room temperature. In fact, a thermoset with a high crosslinking density has a more restricted molecular mobility on cooling and is therefore less able to densely pack in the amorphous glassy state [26,27]. This also means that a matrix with a higher degree of crosslinking will have a larger free volume and consequently worse mechanical properties [26,27]. In other words, a favourable minimum of the reachable free volume will be passed during the conversion process. In addition, a contribution of the ‘rigid filler’ effect is conceivable. In addition to improving the stiffness and general mechanical properties of a material according to the rule of mixture through the use of rigid additives, this effect can reduce the free volume [28,29]. The extent depends on the size of the embedded fillers, their properties, and the interface interactions with the matrix [30].

Applied to the present situation of different mixing ratios, conclusions can also be drawn here about the resulting network structure and the free volume. Starting with region (i), it can be assumed that a wider network will form if a relatively large amount of epoxy is present during the reaction. Accordingly, the fewer available arginine molecules lead to longitudinal and lateral crosslinking. In addition, relatively mobile unreacted or only partially reacted DGEBA molecules are present. These can act as plasticisers on the one hand and increase the mobility of the network on the other so that very dense packing with minimal free volume can form during cooling. For the second region (ii), the crosslinking density increases as the ratio approaches the stoichiometric ratio. Consistently with the increase in steric hindrance during cooling and the resulting higher free volume, the mechanical properties worsen, while T_g_ increases significantly, as shown later in Section 3.5. The third region (iii) then shows the most significant changes from the point where the ratio becomes over-stoichiometric onwards. Here, the formation of a wider network is assumed again as there is not enough epoxy available for the reaction. Particularly, the T_g_ decrease discussed in Section 3.5 and the modulus increase strengthen this hypothesis. However, improvements in the other mechanical properties cannot be observed as they were in the first region (i). In contrast, the tensile strength and elongation at break significantly decrease. The likely absence of a crucial crosslinking reaction with missing epoxy (see Section 4) is mainly a result of the non-reacted arginine particle agglomeration acting as defects in the structure.

**Figure 7:**
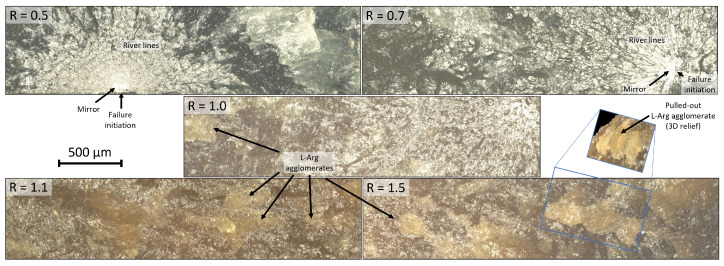
Digital microscopy images of fracture surfaces.

**Figure 8:**
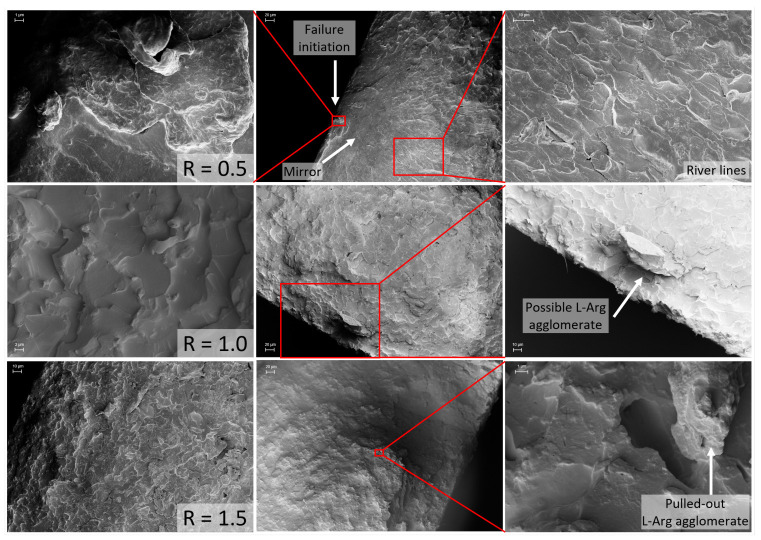
SEM images of fracture surfaces for R = 0.5, R = 1.0, and R = 1.5.

To support the mentioned findings, digital microscopy and SEM images of the fracture surfaces of various configurations are shown in Figure 7 and Figure 8. Firstly, they confirm the observation from the optical examination of increasing light diffraction as a result of increasingly more remaining particles or agglomerates occurring with increasing L-Arg content. Together with the findings from the FTIR investigations, the agglomerated L-Arg particles seem to form strong secondary valence forces (hydrogen bonds) between the unreacted functional -NH and -OH groups of the amino acid molecules, or they might also form peptide bonds. The 3D image and the SEM image show for R = 1.5 that agglomerate pull-outs occur on the fracture surface and that the fracture mechanism differs strongly from the configurations R ≤ 1.0. According to Cantwell et al. [31], these show typical fracture surfaces for brittle materials under tensile loading. After failure initiation (e.g., caused by a defect), a smooth area (mirror) occurs, where crack propagation takes place slowly. Attributed to the generation of secondary cracks in the region of high stress, parabolic profiles (river lines) arise afterwards. Depending on the ratio of the crack propagation rates of primary and secondary cracks, their appearance ranges from parabolic to elliptical. In later stages, a rougher pattern develops, due to the interactions of parabolas [32].

In the theoretical stoichiometric configuration R = 1.0, the formation of agglomerates is already visible but appears to be less critical, since the mechanical properties still remain at a high level.

### 3.5. Glass Transition Temperatures

T_g_ is one of the most relevant thermo-mechanical material parameters of amorphous polymers because it is associated with significant changes in viscoelastic properties. It provides a description of the transition from the glassy state to the rubbery state and is typically correlated with the degree of cure, network formation, and mobility, as well as structural transformations. For example, it gives information on changes in the segmental mobility of polymers [33]. Consequently, T_g_ is relevant for determining the possible service conditions of polymeric or composite parts. According to the literature, different measurement methods yield slightly different values for T_g_, because different processes are analysed: The authors of [25,34] argued that the values for T_g_ differ when the mobility of molecular chains (DMA), the free volume (TMA), or the thermal effects (DSC) are tested. However, Startsev et al. [33] found that parameters (such as the heating rate) also influence the determined T_g_s. However, the equivalence of T_g_s determined with different methods is shown to be achievable.

**Figure 9:**
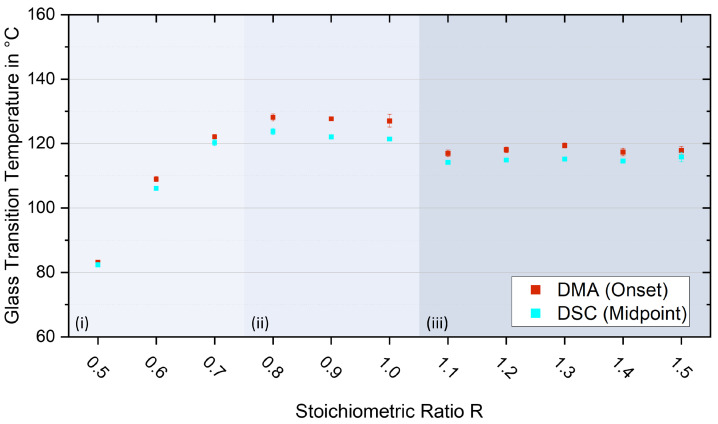
Glass transition temperatures for all investigated mixing ratios analysed via DMA onset (red) resp. DSC midpoint (turquoise) and categorisation into three zones (i–iii).

Since the determination parameters in this study were chosen uniformly for all mixing ratios, the determined T_g_s of the analysed configurations are comparable within each analysis method. Figure 9 shows the T_g_s for all investigated mixing ratios, and the exact values are listed in Table A2 in Appendix A. Generally, values determined using DMA (DMA curves, see Figure 6) and DSC show the same trend.

Importantly, the T_g_s at approximately 127 °C (DMA onset) are the highest for the mechanically preferred ratios between R = 0.8 and R = 1.0 (ii). Here, only marginal changes (ΔT_g_ < 2 °C) can be found. In contrast, significantly reduced T_g_s with a clear decreasing trend are observed with lower curing agent contents. This observation indicates insufficient network formation due to incomplete curing as a result of the lack of the required proportion of hardener. Significant reductions of more than 30 °C can be measured for the configurations R = 0.5 and R = 0.6 (i). For these ratios, much unreacted epoxy remains, as shown in the previous section of the FTIR analysis (blue, see Figure 3). A slightly reduced T_g_ is also evident for R = 0.7, where the epoxy band is already significantly reduced but still observable. All over-stoichiometric configurations (iii) show constant T_g_s of about 118 °C (DMA onset). No decreasing or increasing trend appears with more L-Arg content. Therefore, it can be assumed that the network structure remains basically similar but is hindered by agglomerates of the particulate curing agent added in excess. The formation of agglomerates and peptides is detectable with the results of the FTIR analyses carried out and the optical impressions of the fracture images. In addition, because of the different reactivities of the functional groups, the network density is reduced (see Section 4), as with an increasing amount of hardener, the most reactive groups of the L-Arg molecules react predominantly.

## 4. Postulation of Reaction Mechanism and Resulting Network Structures

Based on FTIR measurements, Li et al. [1] postulated a reaction mechanism for L-Tryptophan-cured epoxies containing an imidazole as a catalyst. Together with the findings published in [3], the results presented in this study can contribute to transferring the proposed mechanism to L-Arg-cured epoxies without a catalyst and postulate a conceivable extension. In [3], rheological measurements and the determination of reaction enthalpy were used to show that the system 827/L-Arg can be stored for a relatively long time at room temperature.

**Figure 10:**
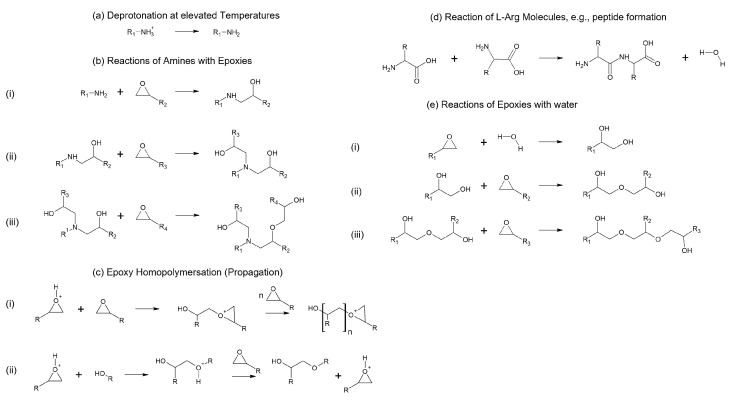
Postulated possible reactions in amino acid-cured epoxies. (**a**) Deprotonation of α-NH_3_^+^ at elevated temperatures allows for (**b**) common amine–epoxy reactions: (**i**) reaction of primary amines; (**ii**) reaction at secondary amine and (**iii**) etherification. (**c**) Catalytic epoxy homopolymerisation: (**i**) ACE mechanism and (**ii**) AM mechanism. (**d**) Amino acids can react to amides (e.g., peptides) and water. (**e**) Water-initiated reactions with epoxies: (**i**) hydrolysis, (**ii**) initiation, and (**iii**) propagation of crosslinking.

Since L-Arg is in the solid state at room temperature and is therefore predominantly/almost exclusively ionised as described in the Introduction [4], it can be assumed that arginine molecules assemble into crystal lattices via these ions [35]. Common amine–epoxy reactions, as shown in Figure 10b, cannot take place in this state because there are no pairs of free electrons available for nucleophilic attack.

It is now assumed that analogously to the catalyst-initiated deprotonation postulated in [1], the α-NH_3_^+^ groups deprotonate when the temperature increases during the curing process (Figure 10a), and the reactions mentioned in Figure 10b can consequently take place. At elevated temperatures, the molecules in the ion lattice tend to vibrate more, presumably allowing deprotonation to occur. It is possible that carboxylates are consequently deionised or that water in the resin (e.g., because of humidity or peptide formation) accepts the proton. As proposed in [1], the formation of primary amines may significantly increase the reactivity of COO^−^, leading to esterification, resulting in the extraction of more hydrogen atoms from the NH_3_^+^ groups. The postulated deprotonation is supported by our own preliminary studies showing that amino acids stabilised with counter-ions, e.g., from hydrochloride (HCl) [36], do not cure epoxides, as the deprotonation of α-NH_3_^+^ is significantly more difficult here.

However, this study demonstrates that the stoichiometric ratio calculated by the number of active hydrogen atoms does not lead to performance-optimised polymers. Instead, this is the case for slightly sub-stoichiometric mixtures.

Taking into account the common epoxy–amine reactions shown in Figure 10b, primary amino groups might react first, as this is energetically favourable in (i), before forming secondary amino groups and hydroxyl groups. The secondary amino groups then react further to form the tertiary (ii) and generate additional -OH groups at the opening of the ring, which can etherify (iii) by opening other oxirane groups [37]. In principle, therefore, an active hydrogen atom can open up more than one oxirane group.

Since the guanidium group is considered very stable in almost all environments [4] due to its mesomeric stabilisation, deprotonation is less likely compared to the α-NH_3_^+^ group. This is comparable to the suggestions of Li et al. [1], because L-Tryptophan includes a nitrogen atom in its indole group delocalised in an aromatic system and was postulated to have a lower reactivity.

Therefore, the number of active hydrogens is drastically reduced with a limited number of epoxide groups, which theoretically would lead to a higher necessary proportion of L-Arg. However, as the results of the thermo-mechanical characterisation tend to indicate a reduced amount of the amino acid as positive, crosslinking via etherification is likely, if sufficient epoxy groups are present. Because of the high temperatures in the curing process, there tends to be enough energy available for these reactions to take place. For the same reason, the homopolymerisation of epoxy groups (c) and amide/peptide formation (d) are also conceivable but not preferred in terms of energy.

The mechanism of possible homopolymerisation is conceivable and may be catalysed by tertiary amines without an additional catalyst due to high curing temperatures [38]. However, a cationic mechanism of homopolymerisation is also plausible due to proton shifts (e.g., due to the proton released by deprotonation of the α-NH_3_^+^ group of L-Arg). In this case, the epoxide oxygen is protonated and becomes more susceptible to nucleophilic attack as a more electrophilic ring. In principle, the propagation of cationic curing, displayed in Figure 10c, can take place via an (i) active chain end (ACE) or (ii) activated monomer (AM) mechanism [39,40]. Compared to amino curing, cationic curing typically produces more linear epoxy chains. Alternatively, the opening of an anionic ring by carboxylate groups (COO^−^) is also theoretically possible. Additional investigations are required to clarify the details.

There are some indications of the formation of the amide/peptide mentioned (d) in the FTIR results presented. Amide formation releases water, which in turn enables the water-induced reactions with epoxides shown in (e) or may accept the protons of the deprotonations.

As no significant epoxy peak is visible above R = 0.7 using FTIR, the epoxy resin still reacts almost completely above this curing agent content, which speaks in favour of the postulated reaction diversity. The non-conclusive list of possible reactions described above explains why the calculation of the stoichiometric ratio is only a guide to find a suitable mixing ratio. However, for some standard systems, e.g., anhydride-cured epoxies, a sub-stoichiometric mixture is also recommended for optimised performance due to the epoxy homopolymerisation that occurs [5].

The postulated mechanisms are also conceivable for other amino acids in solid form. The resp. side chains may be relevant for the following:

(1) The temperatures at which α-NH_3_^+^ is deprotonated: Compared to other amino acids, L-Arg probably requires more energy to deprotonate α-NH_3_^+^ due to the effects of the stable guanidium ion.
(2) The density of the network, for example, due to steric hindrance.
(3) Secondary/valence forces: It is conceivable that, e.g., the ionic bonds of the guanidium ions in L-Arg may improve thermo-mechanical properties.
(4) Additional reactions of the side chains.

Nevertheless, it can be assumed that accelerators or catalysts may change the mechanisms and network structures differently than just lowering the needed energy for deprotonation. Furthermore, other phenomena, such as crystal formation, can occur as reported in [13], which may change mechanisms and the resulting properties.

**Figure 11:**
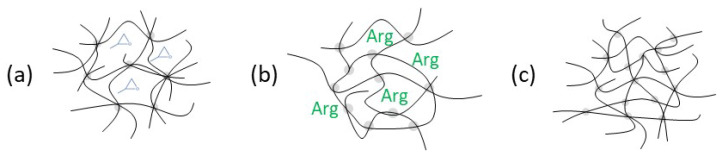
Schematic visualisation of postulated network structures. (**a**) Severely sub-stoichiometric configurations, (**b**) over-stoichiometric configurations, and (**c**) slightly sub-stoichiometric to stoichiometric configurations (with designation of theoretical stoichiometric ratio).

Figure 11 shows schematic visualisations of the postulated network structures for three groups of mixing ratios, explaining the measured thermo-mechanical differences. The configuration R = 0.7 represents a transitional case between (a) and (b) and is therefore not listed below.

### 4.1. SeverelySub-Stoichiometric Configurations R = 0.5–R = 0.6

**Figure A3:**
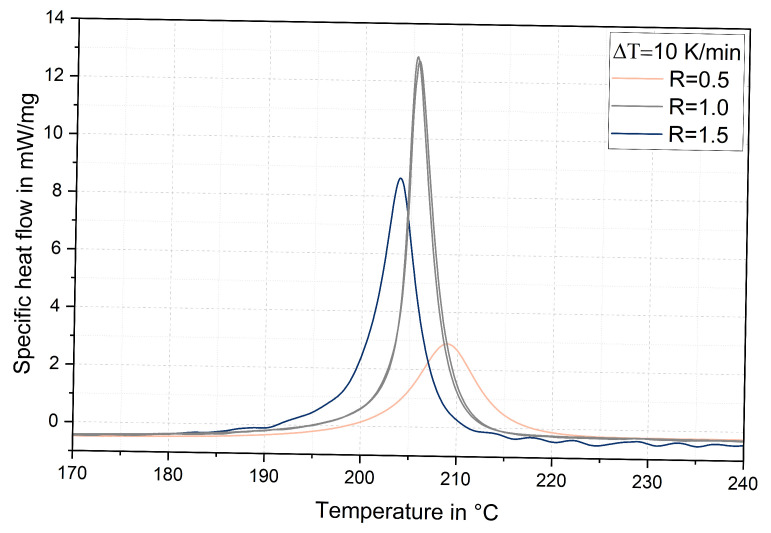
DSC thermograms of curing with heating rates of 10 K/min. Comparison of configurations R = 0.5, R = 1.0, and R = 1.5.

In severely sub-stoichiometric mixtures, a sufficient number of epoxy rings are available to allow all the reactions presented in Figure 10b, including crosslinking through ether bonds (iii), to take place [41]. The significant excess of epoxy probably even deprotonates guanidium ions, since no more peaks are recognisable in FTIR in the 1600–1720 cm^−1^ range. This leads to further crosslinking points. In contrast, homopolymerisation is also possible because of the availability of many epoxy rings, which leads to chain extension between the crosslinking points. There is also the probability that both epoxy rings of the DGEBA molecules will not react because of the lack of curing agent molecules. Consequently, the crosslinking density is reduced at these points. The reaction of L-Arg molecules with each other is unlikely to be favoured due to a lack of availability. Despite all possible reactions, unreacted epoxy groups (detected by FTIR) are still present, which are deposited between crosslinked polymer chains and increase their distance and for this reason have a plasticising effect and, for example, reduce T_g_. For the crosslink density of the configuration R = 0.5, a comparatively low value of $\nu_{C}=0.59\;\cdot \;10^{-3}$ mol · cm^−3^ was determined, which supports the postulated network structure. To further support the assumption that energetically non-preferred reactions also occur, Figure A3 shows the DSC thermogram during curing. For the configuration R = 0.5, the exothermic peak is significantly shifted to higher temperatures compared to mixtures with more L-Arg. Additionally, the evaluated reaction enthalpy is 205.3 J/g, which is approximately half of the enthalpy of the stoichiometric ratio (411.4 J/g).

### 4.2. Over-Stoichiometric Configurations R > 1.0

In over-stoichiometric mixtures, more linear polymer chains are expected, since the reaction in Figure 10b(i) is energetically the most favourable, and the reactions from Figure 10b(ii,iii) and other reactions that require epoxy rings are unlikely due to the lack of epoxy groups, so fewer crosslinks occur. This is in line with the shift in the exothermic peak to lower temperatures in Figure A3. Moreover, arginine-terminated dangling chain ends can be expected [5].

The surplus of arginine molecules may tend to agglomerate (FTIR, microscopy), e.g., because of ionisation. These agglomerates accumulate between chains and cause defect-driven failure and a less dense network (decreasing T_g_). The network density for R = 1.5 ($\nu_{C}\approx 1.54\;\cdot \;10^{-3}$ mol · cm^−3^) is approximately three times higher than that for R = 0.5, which speaks in favour of the discussed structure. Additionally, the evaluated reaction enthalpy (348.5 J/g) is significantly higher. It could not be conclusively clarified whether the additional L-Arg molecules react with each other and form peptide bonds with water cleavage.

### 4.3. Slightly Sub-Stoichiometric to Stoichiometric Configurations R = 0.8–R = 1.0

These DGEBA: L-Arg ratios tend to lead to a complete reaction of the deprotonated amino acid (including crosslinking but without reaction of the guanidium groups) without unreacted molecules reducing the network density. Accordingly, the thermo-mechanical properties are the best in this range. The estimated crosslink density for R = 0.9 ($\nu_{C}\;\approx \;3.0\;\cdot \;10^{-3}$ mol · cm^−3^) and the highest reaction enthalpy (411.4 J/g) underline this outcome.

## 5. Conclusions

In summary, the stoichiometric variations in L-Arg-cured DGEBA epoxy revealed that the range between R = 0.8 and R = 1.0 presents a consistently good trade-off between all the thermo-mechanical properties analysed. The investigated biobased cured epoxy system is comparatively insensitive to variations in the proportion of L-Arg for sub-stoichiometric ratios and offers the possibility of adjusting customised (e.g., optical, thermal, and mechanical) properties via the amount of curing agent. However, in clearly sub-stoichiometric ratios (R ≤ 0.7), the mechanical properties increase, but T_g_ drops significantly. In contrast, any over-stoichiometric ratio immediately reduces mechanical and thermal properties significantly.

The system 827/L-Arg offers a variety of possible reactions due to the functional groups it contains. The change in the mixing ratio causes non-favoured reactions to take place in the case of sub-stoichiometric ratios, which leads to a changed network structure. Therefore, lower proportions of L-Arg (R = 0.5, R = 0.6) cause reduced crosslinking and T_g_s but better mechanical properties, while FTIR spectra show the remaining epoxy groups in significant amounts only for these configurations. However, over-stoichiometric L-Arg contents result in significant reductions in the analysed thermal–mechanical properties mainly because of agglomerate formation. In addition to the different networks formed that lack epoxy groups that allow for crosslinking, this may also be attributed to the possible amidisation/peptidisation of arginine particles, leading to defect-driven failures.

Employing these findings, the authors postulated a complex reaction mechanism for the non-catalysed or non-accelerated arginine-curing of epoxy. As a recommendation, it can be emphasised that slightly sub-stoichiometric ratios should be preferred if the best thermo-mechanical properties need to be achieved while avoiding significant quantities of L-Arg agglomerates. Due to the changing microscopic and macroscopic appearance, the influence of stoichiometric variations on other properties, or long-term behaviour, such as environmental ageing, offers opportunities for future research.

## Figures and Tables

**Table 1:** Mixing ratios of all manufactured configurations.

Stoichiometric Ratio R	827 in g	:	L-Arg in g
0.5	100	:	6.87
0.6	100	:	8.24
0.7	100	:	9.62
0.8	100	:	10.99
0.9	100	:	12.36
1.0	100	:	13.74
1.1	100	:	15.11
1.2	100	:	16.49
1.3	100	:	17.86
1.4	100	:	19.24
1.5	100	:	20.61

## Data Availability

The original contributions presented in this study are included in the article. Further inquiries can be directed to the corresponding author.

## Abbreviations

The following abbreviations are used in this manuscript:

ACE	Active chain end
AHEW	Amine hydrogen equivalent weight
AM	Activated monomer
ATR	Attenuated total reflectance
DGEBA	Bisphenol A diglycidyl ether
DMA	Dynamic mechanical analysis
DSC	Differential scanning calorimetry
EWW	Epoxy equivalent weight
FTIR	Fourier transform infrared spectroscopy
L-Arg	L-arginine
SEM	Scanning electron microscopy
T_g_	Glass transition temperature
TMA	Thermo-mechanical analysis

## Appendix A

**Table A1:** Mechanical properties of all configurations.

Stoichiometric Ratio R	Tensile Strength in MPa	Elongation at Break in %	Young’s Modulus in GPa	Elastic Modulus (DMA) in GPa
0.5	95.81 ± 4.05	4.44 ± 0.54	3.42 ± 0.11	3.61 ± 0.09
0.6	92.04 ± 3.81	5.95 ± 0.72	3.09 ± 0.12	3.25 ± 0.00
0.7	88.99 ± 3.05	7.05 ± 0.76	2.79 ± 0.09	2.99 ± 0.07
0.8	90.17 ± 1.27	7.16 ± 0.34	2.81 ± 0.15	2.90 ± 0.02
0.9	87.87 ± 3.78	6.17 ± 0.51	2.80 ± 0.13	2.93 ± 0.01
1.0	87.31 ± 1.40	5.97 ± 0.75	2.90 ± 0.11	2.97 ± 0.01
1.1	72.24 ± 2.02	3.30 ± 0.23	2.92 ± 0.08	3.09 ± 0.00
1.2	74.82 ± 1.89	3.55 ± 0.19	3.01 ± 0.10	3.05 ± 0.03
1.3	74.69 ± 4.53	3.45 ± 0.30	3.07 ± 0.08	3.16 ± 0.00
1.4	73.86 ± 2.42	3.54 ± 0.25	2.98 ± 0.05	3.12 ± 0.05
1.5	73.25 ± 2.83	3.11 ± 0.09	3.27 ± 0.10	3.19 ± 0.00

**Table A2:** Glass transition temperatures of all configurations.

Stoichiometric Ratio R	T_g_ DSC Midpoint in °C	T_g_ DMA Onset in °C
0.5	82.35 ± 0.75	83.11 ± 0.29
0.6	106.10 ± 0.30	108.95 ± 0.76
0.7	120.30 ± 1.00	122.09 ± 0.72
0.8	123.70 ± 1.00	128.13 ± 1.10
0.9	122.05 ± 0.05	127.65 ± 0.41
1.0	121.35 ± 0.35	127.09 ± 1.97
1.1	114.15 ± 0.05	116.96 ± 1.03
1.2	114.85 ± 0.25	118.05 ± 0.85
1.3	115.15 ± 0.15	119.38 ± 0.78
1.4	114.55 ± 0.15	117.34 ± 1.17
1.5	115.80 ± 1.50	117.81 ± 1.22
